# Schizophrenia and catatonia: from ICD-10 to ICD-11

**DOI:** 10.1007/s00115-025-01861-3

**Published:** 2025-09-04

**Authors:** T. Nickl-Jockschat, J. Steiner, D. Hirjak, A. Hasan

**Affiliations:** 1 Department of Psychiatry and Psychotherapy, Otto-von-Guericke-University Magdeburg, Leipziger Str. 44, 39120 Magdeburg, Germany; 2 German Center for Mental Health (DZPG), Site Halle-Jena-Magdeburg, Magdeburg, Germany; 3 Department of Psychiatry, Iowa Neuroscience Institute, Department of Neuroscience and Pharmacology, University of Iowa, Iowa City, USA; 4 Department of Psychiatry and Psychotherapy, Central Institute for Mental Health, Medical Faculty Mannheim, University of Heidelberg, Mannheim, Germany; 5 German Center for Mental Health (DZPG), Site Mannheim-Heidelberg-Ulm, Mannheim, Germany; 6 Department of Psychiatry, Psychotherapy and Psychosomatics, Medical Faculty, University of Augsburg, Augsburg, Germany; 7 German Center for Mental Health (DZPG), Site München/Augsburg, Augsburg, Germany

**Keywords:** Psychosis, Classification, Diagnostic criteria, Categorical-dimensional model, Psychomotor symptoms, Psychose, Klassifikation, Diagnostische Kriterien, Kategorial-dimensionaler Ansatz, Psychomotorik

## Abstract

The classification of psychotic disorders has undergone a variety of changes. Since Karl Ludwig Kahlbaum’s (Kahlbaum 1874) first descriptions of catatonic states and Emil Kraepelin’s (Kraepelin 1883) nosological classification of psychotic syndromes in the second half of the nineteenth century, the diagnostic criteria for these disorders have been repeatedly modified, significantly impacting clinical practice. Eugen Bleuler (Bleuler 1911) coined the term “schizophrenia”, emphasizing the disturbances in thinking, feeling and acting that he had observed. With the introduction of the 11th version of the International Classification of Diseases (ICD-11), several significant changes to the diagnostic criteria were introduced. First-line symptoms according to Schneider lost importance. The subtypes (e.g., paranoid, hebephrenic and catatonic schizophrenia) were also omitted and symptom and progression classifiers have been introduced instead. Finally, catatonia is now defined as an independent diagnostic entity, while in ICD-10 it was still assigned to schizophrenia under the code F20.2. This recognizes catatonia’s independent, cross-diagnostic nature. Due to these symptom and progression classifiers, the ICD-11 now takes a more a hybrid categorical and dimensional approach to the diagnosis than the previous version.

## Abstract (de)

Die Klassifikation der Erkrankungen des schizophrenen Formenkreises hat einen vielfältigen Wandel durchlaufen. Seit den ersten Beschreibungen katatoner Zustandsbilder durch Karl Ludwig Kahlbaum (Kahlbaum, 1874) sowie der nosologischen Einordnung psychotischer Syndrome durch Emil Kraepelin (Kraepelin, 1883) in der zweiten Hälfte des 19. Jahrhunderts kam es immer wieder zu Veränderungen in den Diagnosekriterien dieser Störungsbilder mit entsprechenden Folgen für die klinische Praxis. Eugen Bleuler (Bleuler, 1911) prägte den Begriff Schizophrenie und betonte damit die von ihm beobachteten Spaltungsphänomene im Denken, Fühlen und Handeln. Mit der Einführung der 11. Version der International Statistical Classification of Diseases and Related Health Problems (ICD-11) kam es zu mehreren signifikanten Veränderungen. Zum einen verloren die Erstrangsymptome an Bedeutung. Auch wurden die Subtypen (z. B. paranoide, hebephrene, katatone Schizophrenie) abgeschafft, stattdessen wurden Symptom- und Verlaufsklassifikatoren eingeführt. Schließlich wurde die Katatonie nun als eigenständige diagnostische Entität definiert, während sie in der ICD-10 noch unter dem Code F20.2 den Schizophrenien zugeordnet war. Damit wird dem eigenständigen, diagnoseübergreifenden Ansatz der Katatonie Rechnung getragen. Durch die zuvor genannten Symptom- und Verlaufsklassifikatoren setzt die ICD-11 im Vergleich zur Vorversion verstärkt auf einen hybriden kategorial-dimensionalen Ansatz zur Diagnosestellung.

## Introduction

The diagnostic criteria for schizophrenia and catatonic syndromes have undergone numerous conceptual and nosological changes throughout history—most recently, in a substantial way, with the introduction of the *Eleventh Revision of the International Classification of Diseases* (ICD-11). In this article, we examine the key innovations of the ICD-11 regarding the classification of schizophreniform disorders and catatonia. The focus lies on the abandonment of traditional subtypes, the introduction of symptom and course specifiers, and the reevaluation of catatonia as an independent, cross-diagnostic entity (see Table 1). The group formerly referred to in ICD-10 as “Schizophrenia, schizotypal, and delusional disorders” has been renamed “Schizophrenia or other primary psychotic disorders” in ICD-11.

**Table 1:** Comparison of the diagnostic groups “Schizophrenia, schizotypal and delusional disorders” in ICD-10 and “Schizophrenia or other primary psychotic disorders” in ICD-11

ICD-10	ICD-11 (Draft version)
Schizophrenia, schizotypal and delusional disorders	Schizophrenia and other primary psychotic disorders
Paranoid schizophrenia (F20.0)	Schizophrenia, first episode (6A20.0)
Hebephrenic schizophrenia (F20.1)	Schizophrenia, multiple episodes (6A20.1)
Undifferentiated schizophrenia (F20.3)	Schizophrenia, continuous (6A20.2)
Postschizophrenic depression (F20.4)	–
Residual schizophrenia (F20.5)	–
Schizophrenia simplex (F20.6)	–
Other schizophrenia (F20.8)	Other specified episode of schizophrenia (6A20.Y)
Schizophrenia, unspecified (F20.9)	Schizophrenia, episode unspecified (6A20.Z)
Schizotypal disorder (F21)	Schizotypal disorder (6A22)
Persistent delusional disorders (F22)	Delusional disorder (6A24)
Induced delusional disorders (F24)	
Acute polymorphic psychotic disorders (F23)	Acute and transient psychotic disorders (6A23)
Schizoaffective disorders (F25)	Schizoaffective disorders (6A21)

For catatonic schizophrenia (F20.2), please refer to Table 7

## Diagnostic criteria for schizophrenia according to ICD-10

According to ICD-10, a diagnosis of schizophrenia was made based on characteristic constellations of symptoms that persisted for a period of at least 1 month. ICD-10 distinguished between two complexes of four symptoms each:

At least one unambiguous symptom from groups one to four (or two or more if less unambiguous) over a period of at least 1 month:

1. Thought echo; thought insertion or withdrawal; thought broadcasting
2. Delusions of control or influence; experiences of passivity in relation to body movements, thoughts, actions, or sensations; delusional perception
3. Hallucinatory voices that provide a running commentary on the patient or having a dialog about the patient among themselves
4. Persistent culturally inappropriate, bizarre delusions

Or at least two clear symptoms from groups five to eight over a period of at least 1 month:

5. Persistent hallucinations in any sensory modality
6. Neologisms, thought blocking or insertion into the train of thought
7. Catatonic symptoms (excitement, posturing, negativism, etc.)
8. Negative symptoms such as poverty of speech, affective flattening, or apathy

## ICD-11 diagnostic criteria for schizophrenia (code: 6A20)

In contrast to the old concept, diagnosis under ICD-11 is based on a combined (or hybrid) categorical–dimensional approach. The full criteria can be found on the WHO website (https://icd.who.int/en).

### Core diagnosis code (categorical approach)

When the categorical threshold is met (two symptoms, at least one of which is a core symptom), with a duration of ≥ 1 month and exclusion of other causes (differential diagnosis), schizophrenia can be diagnosed. The ICD-11 task group had explored arguments for harmonizing the minimum required symptom duration with the DSM‑5 (≥ 6 months), but due to lack of evidence favoring one duration over the other, the ICD-11 retained the 1‑month criterion from ICD-10.

Thus, as in ICD-10, the following symptoms must be present for at least 1 month to diagnose schizophrenia.

#### Core symptoms

At least two symptoms must be present from a list of seven categories. At least one must stem from the so-called core symptoms (a–d):

- *Delusions* (persistent delusional beliefs): Examples given in ICD-11 include persecutory, grandiose, or referential delusions.
- *Hallucinations* (persistent hallucinations): Auditory hallucinations are most common, but they can occur in any sensory modality.
- *Disorganized thinking*: Typically manifests as formal thought disorder—e.g., loose associations, neologisms, incoherence, or “word salad.”
- *Experiences of influence, passivity, or control*: ICD-11 describes this as the experience that emotions, impulses, thoughts, bodily functions, or behavior are being controlled by an external force—e.g., thought insertion, withdrawal, or broadcasting.

#### Additional symptoms (e–g)

e) *Negative symptoms* (e.g., affective flattening, poverty of speech, lack of motivation)
f) *Disorganized behavior* (e.g., bizarre or purposeless behavior, unpredictable or inappropriate emotional responses)
g) *Psychomotor disturbances* (including catatonic symptoms such as catatonic excitement)

##### Note.

In ICD-11, catatonia is classified as an independent disorder, no longer as a subtype of schizophrenia. If catatonic symptoms are present and diagnostic criteria are met (see below), a separate diagnosis of catatonia should be coded in addition to schizophrenia (Code 6A40: Catatonia associated with another mental disorder).

#### Exclusion of other causes

As in ICD-10, symptoms must not be attributable to other medical conditions, substance use, or mood disorders; ICD-11 remains somewhat vague on this. In clinical practice, it is therefore advisable to follow the recommendations of the AWMF/DGPPN S3 Guideline on Schizophrenia [1] for organic initial, differential, and course diagnostics [2]. The guideline adopts a clinically pragmatic approach based on “red flag” symptoms that indicate an organic cause and necessitate further diagnostics (e.g., lumbar puncture, rheumatological labs, EEG, etc.). In general, it is recommended that—unless contraindicated—every person with schizophrenia undergoes magnetic resonance imaging (MRI) of the brain at least once in their lifetime. Secondary psychotic syndromes will be coded in the future as 6E61.1.

### Extension codes (dimensional approach)

The former subtypes (paranoid, hebephrenic, catatonic, etc.) have been removed. Instead, course and symptom specifiers can now be used.

#### Course specifiers

The episode status is integrated into the main code, as the first digit after the period (Table 2).

**Table 2:** Episode status as a course specifier of schizophrenia

Code	Significance
6A20.*0*	Schizophrenia, first episode
6A20.*1*	Schizophrenia, multiple episodes
6A20.*2*	Schizophrenia, continuous
6A20.*Y*	Other symptoms
6A20.*Z*	Not further specified

The *remission status* is also integrated into the main code, as the second digit after the period (Table 3).

**Table 3:** Remission status as a course specifier of schizophrenia

Code	Significance
6A20.x*0*	Currently symptomatic
6A20.x*1*	In partial remission
6A20.x*2*	In full remission
6A20.x*Z*	Unspecified

*Example: *6A20.10 = schizophrenia, multiple episodes, currently symptomatic

#### Symptom classifiers in the dimensional concept

Symptom classifiers are listed in Table 4.

**Table 4:** Schizophrenia symptom classifiers

Code	Symptom dimension
6A25.0	Positive symptoms
6A25.1	Negative symptoms
6A25.2	Depressive symptoms
6A25.3	Manic symptoms
6A25.4	Psychomotor symptoms
6A25.5	Cognitive symptoms

#### Severity classifiers

These are applied as extension codes (e.g., *XS0T, XS5W*) and are post-coordinated to the main code. They describe the intensity of the symptom dimension, independently of the disorder itself (Table 5).

**Table 5:** Schizophrenia severity classifiers

Code	Severity
XS8H	No symptoms
XS5W	Mild symptoms
XS0T	Moderate symptoms
XS25	Severe Symptoms

##### Infobox ICD-11 diagnostic criteria of schizophrenia

The ICD-11 diagnostic criteria for schizophrenia (WHO) explicitly require:

- At least *two symptoms* from a list of *seven symptom domains.*
- At least *one* must come from *points (a) through (d).*
- *Duration*: Symptoms must be present “*most of the time over a period of ≥* *1 month.*”

Thus, the criteria do *not* represent a purely open dimensional approach, but rather a *hybrid form*:

- *Categorical threshold*: (two symptoms, at least one from a–d)
- *Dimensional additions*: (symptom qualifiers, severity, functional impact, course)

## Changes in ICD-11 compared to previous ICD-10 criteria for schizophrenia

The diagnosis of schizophrenia in ICD-11 is now based on a more modern, holistic, and hybrid *categorical–dimensional approach*, which weighs various symptom clusters (positive, negative, disorganized, cognitive, and catatonic symptoms) in relation to functional status, severity of impairment, and individual course of illness. This replaces the rigid classification into specific subtypes as in ICD-10 in favor of a more *flexible and individualized diagnostic process*. ICD-11 aims to better account for the individual trajectory of the illness rather than relying solely on the presence of a fixed number of specific symptoms. The subtypes used in ICD-10 have also been eliminated (see below).

Notably, *catatonia is now classified as an independent diagnosis*, and no longer part of the schizophrenia spectrum.

## ICD-11 diagnostic criteria for catatonia

The new ICD-11 classification marks a *paradigmatic shift* in the diagnosis of catatonia: For the first time since Karl Kahlbaum’s original description, it once again centers on the *psychomotor nature* of the syndrome—departing from the century-long interpretation initially described by Emil Kraepelin and Eugen Bleuler [3, 4]. ICD-11 thus enables *syndrome-oriented diagnostics* and facilitates the distinction from clinically similar conditions such as *neuroleptic malignant syndrome (NMS)* or delirium.

Catatonia according to ICD-11 can be diagnosed based on *15 clinical signs and symptoms* (see Table 6), categorized into three groups of psychomotor abnormalities: *reduced, increased*, and *abnormal* psychomotor activity [5]. This classification reflects research findings that differentiate between *hypokinetic, hyperkinetic*, and *parakinetic* forms [6, 7].

**Table 6:** Symptoms of catatonia according to ICD-11

Reduced psychomotor activity	Increased psychomotor activity	Abnormal psychomotor activity
Staring	Extreme hyperactivity or agitation without cause, with non-purposeful movements	Grimacing
Ambitendency	Uncontrollable, extreme emotional reactions	Mannerisms
Negativism	Impulsivity	Posturing
Stupor	Aggression toward others	Stereotypies
Mutism		Rigidity
		Echolalia/Echopraxia
		Verbigeration
		Waxy flexibility
		Catalepsy

Interestingly, these criteria are unevenly distributed:

- *Nine features* are assigned to the *abnormal* group.
- *Five features* to the *reduced* group.
- And *only one combined criterion* for *increased* psychomotor activity.

Although this domain is formally counted as a *single criterion*, it actually includes several clinically relevant phenomena: extreme hyperactivity or agitation without obvious cause, purposeless movements, and/or uncontrollable extreme emotional reactions; *impulsivity* (sudden, unprovoked inappropriate behavior); and *aggression toward others*, with or without risk of injury. Despite this symptom diversity, the simultaneous presence of several of these forms is counted only as *one criterion*, unlike the other two symptom groups [5].

As a result, *purely hypokinetic or parakinetic* forms of catatonia can be diagnosed based on their symptoms, whereas *hyperkinetic catatonia* requires *additional symptoms from the other two groups* ([5]; see Table 7).

**Table 7:** The newly established diagnostic group of catatonia in ICD-11 and the previously classified diagnosis of catatonic schizophrenia in ICD-10

ICD-10	ICD-11 (Draft version)
	Catatonia
Catatonic schizophrenia (F20.2)	Catatonia associated with another mental disorder (6A40)
–	Catatonia induced by substances or medications (6A41)
–	Secondary catatonia syndrome (6E69)
–	Catatonia, unspecified (6A4Z)

Another diagnostically relevant ICD-11 criterion for catatonia concerns *duration*. Typically, signs must persist for *several hours* to be considered diagnostically significant. However, in cases of particularly striking symptoms—such as *stupor, catalepsy, mutism*, or *negativism*—or when accompanied by *autonomic disturbances* (e.g., cardiovascular or respiratory dysregulation), a *shorter duration of around 15* *min* may suffice. This *temporal flexibility* reflects clinical reality, where catatonic states may be acute and transient.

ICD-11 also explicitly notes that catatonia can occur *across the lifespan*, consistent with evidence that catatonic syndromes can be observed in *childhood and adolescence *[8, 9]. At the same time, catatonia represents an important *differential diagnosis in older adults*, making it a relevant clinical phenomenon for both *child/adolescent psychiatry* and *geriatric psychiatry *[10].

## ICD-11 diagnostic subcategories for catatonia

- *Catatonia associated with other mental disorders (Code 6A40):* Can occur with conditions such as schizophrenia, developmental disorders, bipolar disorder, or depression.
- *Catatonia induced by substances or medications (Code 6A41):* A distinct diagnostic category for catatonic syndromes associated with intoxication or withdrawal.
- *Secondary catatonia syndrome/catatonia due to medical conditions (Code 6E69):* Allows for a diagnosis in the context of neurological or other physical illnesses. Conditions that can be associated include: diabetic ketoacidosis, hypercalcemia, hepatic encephalopathy, homocystinuria, neoplasms, head trauma, cerebrovascular disease, and encephalitis. In cases of *early-onset, acute psychosis* or *rapid deterioration of a developmental disorder*, especially with *focal neurological symptoms, autoimmune encephalitis* (e.g., anti-NMDAR) must be ruled out.
- *Unspecified catatonia (Code 6A4Z):* A residual category when no specific cause can be assigned.

With catatonia now recognized as an independent diagnosis, ICD-11 better reflects the *etiological and clinical heterogeneity* of catatonic syndromes. By clearly separating it from schizophrenia, it brings both *conceptual clarity* and *therapeutic relevance* to clinical practice: *Antipsychotics are no longer considered the standard treatment* for catatonia. On the contrary, they may *worsen symptoms, trigger neuroleptic malignant syndrome*, or *obscure diagnosis* due to extrapyramidal side effects [11]. While antipsychotic use may be considered in specific cases—such as catatonic states within psychotic episodes—*caution is essential*. ICD-11 provides *critical therapeutic flexibility*, allowing for targeted treatment with *benzodiazepines* or *electroconvulsive therapy (ECT) independent* of a schizophrenia diagnosis. Future research should focus on identifying which symptoms most *specifically and reliably* indicate catatonic states. Additionally, *standardization of catatonic signs and symptoms* in both research and clinical settings is desirable to further improve *diagnostic clarity* and *international comparability*.

## Practical conclusion

- ICD-11 *replaces the previous subtype system* of schizophrenia according to ICD-10 with a *hybrid categorical–dimensional approach*, integrating symptom and course specifiers into diagnosis.
- *Categorical*: Diagnosis of schizophrenia requires *at least two symptoms*, one of which must be from the core group (delusions, hallucinations, thought disorder, disturbances of self-experience) present for *at least 1 month*.
- *Dimensional*: Symptom and course specifiers (e.g., remission status, dominant symptom dimension such as negative, psychomotor, or cognitive) can be coded additionally.
- ICD-11 thus marks a departure from the historical emphasis on *first-rank symptoms* per Kurt Schneider.
- *Catatonia is no longer a schizophrenia subtype* but an *independent, cross-diagnostic entity* with specific ICD-11 criteria.
- The new catatonia classification includes symptoms from three *psychomotor domains* (reduced, increased, abnormal) and eliminates the 1‑month duration requirement from ICD-10, also accounting for *shorter-lasting states*.
- The ICD-11 catatonia diagnosis represents a *clinically meaningful advancement*, recognizing catatonic syndromes *outside of schizophrenia* and harmonizing diagnostic criteria *regardless of underlying condition*.
- *Catatonic syndromes can now be diagnosed more precisely*, independently of the underlying illness—whether *affective, developmental, substance-related, or medical*.
- This opens new *diagnostic and therapeutic options*: Catatonia can be treated *independently of a schizophrenia diagnosis*, e.g., with *benzodiazepines* or *ECT*, while antipsychotics should be used *with caution*.
- Overall, ICD-11 offers *enhanced opportunities* for *refined diagnostics* and initiation of *tailored, individualized treatments*.

## Declarations

For this article no studies with human participants or animals were performed by any of the authors. All studies mentioned were in accordance with the ethical standards indicated in each case.

The supplement containing this article is not sponsored by industry.
